# Inhibitory Effect of Bacterial Lysates Extracted from *Pediococcus acidilactici* on the Differentiation of 3T3-L1 Pre-Adipocytes

**DOI:** 10.3390/ijms231911614

**Published:** 2022-10-01

**Authors:** Han Bin Lee, Seok-Seong Kang

**Affiliations:** Department of Food Science and Biotechnology, College of Life Science and Biotechnology, Dongguk University-Seoul, Goyang 10326, Korea

**Keywords:** postbiotics, bacterial lysates, *Pediococcus acidilactici*, adipogenesis

## Abstract

Postbiotics, including bacterial lysates, are considered alternatives to probiotics. The aim of the current study was to investigate the effect of bacterial lysates (BLs) extracted from *Pediococcus acidilactici* K10 (K10 BL) and *P. acidilactici* HW01 (HW01 BL) on the differentiation of 3T3-L1 pre-adipocytes. Both K10 and HW01 BLs significantly reduced the accumulation of lipid droplets and the amounts of cellular glycerides in 3T3-L1 cells (*p* < 0.05). However, another postbiotic molecule, peptidoglycan of *P. acidilactici* K10 and *P. acidilactici* HW01, moderately inhibited the accumulation of lipid droplets, whereas heat-killed *P. acidilactici* did not effectively inhibit the lipid accumulation. The mRNA and protein levels of the transcription factors, peroxisome proliferator-activated receptor γ and CCAAT/enhancer-binding protein α, responsible for the differentiation of 3T3-L1 cells, were significantly inhibited by K10 BL and HW01 BL (*p* < 0.05). Both K10 and HW01 BLs decreased adipocyte-related molecules, adipocyte fatty acid-binding protein and lipoprotein lipase, at the mRNA and protein levels. Furthermore, both K10 and HW01 BLs also downregulated the mRNA expression of leptin, but not resistin. Taken together, these results suggest that *P. acidilactici* BLs mediate anti-adipogenic effects by inhibiting adipogenic-related transcription factors and their target molecules.

## 1. Introduction

It has been recognized that an essential concept of probiotics is the viability of microbial cells to ensure beneficial effects for the host. However, recent studies have shown that microbial viability is not necessary when conferring probiotic effects to achieve health promotion, because some mechanisms and clinical benefits are not directly associated with the live microorganisms [1]. In addition, studies have highlighted the limitations of probiotics such as strain-specific behavior, virulence gene transfer, and opportunistic infections [2]. Therefore, new terms, such as postbiotics, have emerged, which indicate that microbial viability is not required for their health-promoting effects [3].

Postbiotics, including cell wall fragments, exopolysaccharides, enzymes, short-chain fatty acids, and bacterial lysates, refer to soluble factors secreted by live bacteria or released after bacterial lysis [3,4]. Furthermore, heat-killed bacteria that demonstrate health benefits also fall into the scope of postbiotics [5]. Several reports have suggested that postbiotic molecules have various functional properties, such as antimicrobial, antioxidant, anti-obesogenic, and immunomodulatory activities [6,7,8,9]. Among the postbiotic molecules, bacterial lysates (BLs) of *Enterococcus lactis* and *Lactobacillus acidophilus* obtained from their degradation products by chemical and mechanical treatments alleviated drug-induced liver injury [10]. Moreover, BLs of *L. amylovorus* reduced the levels of triglycerides and LDL cholesterol in obese mice [9]. 

Adipogenesis is a complex multi-step process of the proliferation and differentiation of pre-adipocytes into mature adipocytes containing lipid droplets, which contribute to obesity [11]. The differentiation of pre-adipocytes into adipocytes is accompanied by specific transcription factors, including peroxisome proliferator-activated receptor γ (PPARγ), CCAAT/enhancer-binding proteins (C/EBPs), and sterol regulatory element binding-proteins 1 (SREBP-1) [12,13]. The differentiation of 3T3-L1 pre-adipocytes into mature lipid-containing adipocytes is commonly used as an in vitro model to assess the molecular characteristics of adipogenesis [14]. During the process of pre-adipocyte differentiation, the temporal expressions of C/EBPβ and C/EBPδ facilitate the expression of the key adipocyte transcription factors, PPARγ and C/EBPα, which are responsible for the maturation and differentiation of adipocytes [15,16]. This is then followed by the enhanced expression of genes that characterize adipogenic phenotypes, including adipocyte-specific fatty acid binding protein (aP2) and leptin [17].

Several in vitro studies have shown that live bacteria and postbiotic molecules from *Lactobacillus* have anti-adipogenic effects [18,19,20,21]. Exopolysaccharides isolated from *L. rhamnosus* GG inhibit the differentiation of 3T3-L1 pre-adipocytes [19]. Cell extracts from *L. plantarum* KY1032 reduce lipid accumulation in 3T3-L1 cells and downregulate adipogenic-related genes, including PPARγ and C/EBPα [20]. Pediococci are Gram-positive lactic acid bacteria commonly found in fermented vegetables, dairy products, and meats [22,23]. *Pediococcus acidilactici* is a potential probiotic that has antimicrobial, antioxidant, and immunomodulatory activities [24,25,26]. Although the oral administration of live *P. acidilactici* M76 decreased body weight and adipose tissue weight in high fat diet-induced obese mice as well as reducing total cholesterol and triglycerides [27], the anti-adipogenic effects of postbiotic molecules from *P. acidilactici* have not been studied. In addition, the inhibitory effects of *P. acidilactici* postbiotic molecules on the differentiation of 3T3-L1 pre-adipocytes and the associated intracellular mechanisms have not been demonstrated. Therefore, in this study, we investigated the anti-adipogenic effects of BLs extracted from *P. acidilactici* K10 and *P. acidilactici* HW01 on the differentiation of 3T3-L1 pre-adipocytes. Moreover, the intracellular mechanisms related to adipogenesis were also investigated.

## 2. Results and Discussion

### 2.1. P. acidilactici BLs Reduce Lipid Accumulation and Cellular Triglycerides in 3T3-L1 Cells

**Figure 1:**
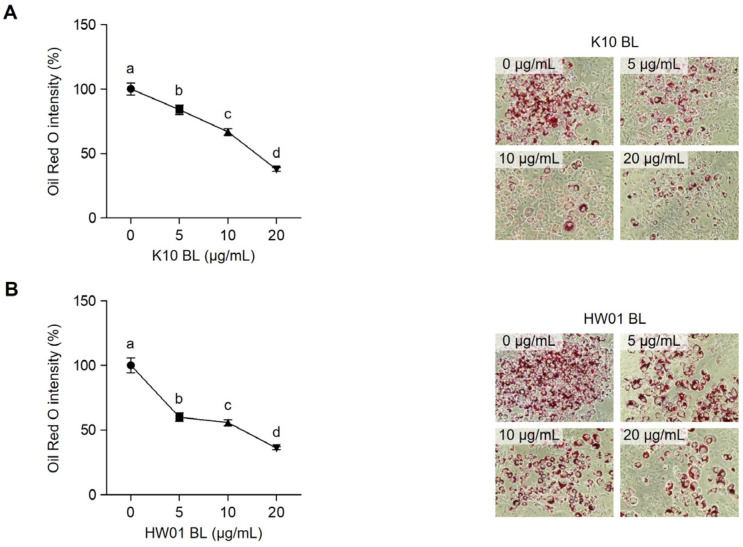
Inhibitory effect of *P. acidilactici* BLs on lipid accumulation in 3T3-L1 cells. Cells were treated with various concentrations (0, 5, 10, and 20 μg/mL) of *P. acidilactici* K10 BL (**A**) or *P. acidilactici* HW01 BL (**B**). At day 12, lipid accumulation in 3T3-L1 cells was assessed by Oil Red O staining. Representative microscopic images of Oil Red O-stained cells are shown. Data are expressed as the mean ± standard deviation. Statistical significance between groups was determined by ANOVA when *p* < 0.05. Significant differences between treatment with K10 BL or HW01 BL are expressed with different letters (a, b, c, d).

**Figure 2:**
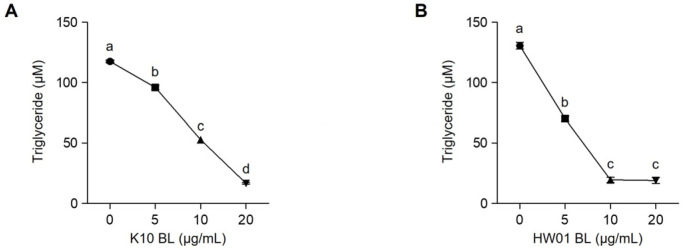
Inhibitory effect of *P. acidilactici* BLs on cellular triglycerides in 3T3-L1 cells. Cells were treated with various concentrations (0, 5, 10, and 20 μg/mL) of *P. acidilactici* K10 BL (**A**) or *P. acidilactici* HW01 BL (**B**). At day 12, the amounts of cellular triglyceride were assessed using a commercial triglyceride assay kit. Data are expressed as the mean ± standard deviation. Statistical significance between groups was determined by ANOVA when *p* < 0.05. Significant differences between treatment with K10 BL or HW01 BL are expressed with different letters (a, b, c, d).

As shown in Figure 1A, the lipid accumulation in 3T3-L1 cells was dose-dependently decreased by the treatment with *P. acidilactici* K10 BL (K10 BL). The treatment with 5 μg/mL of K10 BL reduced the lipid accumulation by 16%. A further reduction of lipid accumulation was observed when the cells were treated with 10 or 20 μg/mL (33% and 62% reduction, respectively). Similarly, *P. acidilactici* HW01 BL (HW01 BL) decreased the lipid accumulation in a dose-dependent manner. The extent of lipid accumulation in 3T3-L1 cells in the presence of HW01 BL decreased by 39%, 44%, and 64% at concentrations of 5, 10, and 20 μg/mL, respectively (Figure 1B). The impact of BLs on the reduction of cellular triglycerides was also determined. Figure 2A shows that the amounts of cellular triglycerides were decreased in the presence of K10 BL in a dose-dependent manner. At a high concentration of K10 BL (20 μg/mL), the extent of cellular triglycerides was markedly decreased by 85%. At the lower concentrations (5 and 10 μg/mL), K10 BL also significantly reduced the amounts of cellular triglycerides (18% and 55% reduction, respectively). Moreover, HW01 BL also significantly reduced the amounts of cellular triglycerides (Figure 2B). Almost 46% of cellular triglycerides were reduced by the treatment with 5 μg/mL of HW01 BL and a further decrease in cellular triglycerides was observed at 10 and 20 μg/mL of HW01 BL (both 85% reduction, respectively). These results suggest that *P. acidilactici* BLs potently inhibited adipogenesis. Lactic acid bacteria, in particular *Lactobacillus*, have been studied as preventive and therapeutic agents against obesity in animal studies and clinical trials [28]. In this study, *P. acidilactici* BLs effectively inhibited adipogenesis by reducing lipid accumulation and cellular triglycerides. In addition to probiotics, the beneficial effects of lysates derived from probiotics have been reported. Lysates of *L. casei* attenuate colitis [29]. The antitumor activity of *L. acidophilus* lysates has been also reported [30]. Moreover, cell lysates from probiotics including lactobacilli lower the cholesterol level, whereas the intact cells of these probiotics do not effectively reduce the cholesterol level in comparison with the cell lysates [31]. Although the beneficial effects of lysates from lactobacilli have been well documented, the health promoting properties, such as anti-adipogenic effect, of lysates from *P. acidilactici* have not been suggested. Furthermore, although a specific component(s) of *P. acidilactici* BLs associated with the inhibition of 3T3-L1 cell differentiation has not been determined in the current study, it can be possibly explained that specific peptide(s) in *P. acidilactici* BLs is properly involved in the anti-adipogenic effect. A previous report has shown that two peptides, LLRLTDL and GYALPCDCL, were revealed to be highly effective for the inhibition of lipid accumulation in adipocytes [32], suggesting that peptides in *P. acidilactici* BLs possibly inhibit 3T3-L1 cell differentiation. Although extensive studies are required to identify specific component(s) in *P. acidilactici* BLs that are closely associated with the anti-adipogenic effect, this study highlights the inhibitory potential of *P. acidilactici* BLs in the prevention of adipogenesis.

### 2.2. P. acidilactici PGN and Heat-Killed P. acidilactici Do Not Effectively Reduce Lipid Accumulation in 3T3-L1 Cells

**Figure 3:**
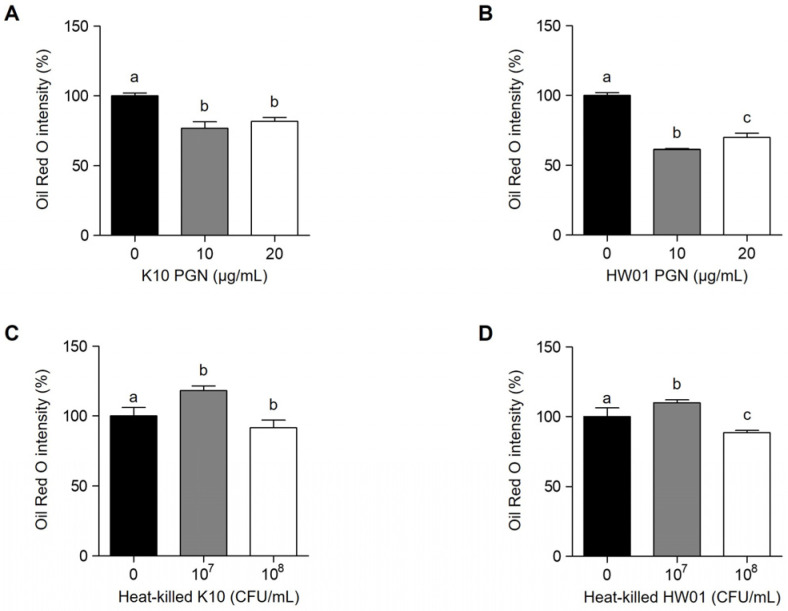
Effect of *P. acidilactici* PGNs and heat-killed *P. acidilactici* on lipid accumulation in 3T3-L1 cells. Cells were treated with various concentrations (0, 10, and 20 μg/mL) of *P. acidilactici* K10 PGN (**A**) or *P. acidilactici* HW01 PGN (**B**). Cells were treated with various concentrations (0, 10^7^ and 10^8^ CFU/mL) of heat-killed *P. acidilactici* K10 (**C**) or *P. acidilactici* HW01 (**D**). At day 12, lipid accumulation in 3T3-L1 cells was assessed by Oil Red O staining. Data are expressed as the mean ± standard deviation. Statistical significance between groups was determined by ANOVA when *p* < 0.05. Significant differences between treatment with *P. acidilactici* PGN (**A**,**B**) or heat-killed *P. acidilactici* (**C**,**D**) are expressed with different letters (a, b, c).

To further examine whether other postbiotic molecules, *P. acidilactici* PGN, a major cell wall component, and heat-killed *P. acidilactici*, have anti-adipogenic effects, the lipid accumulation in 3T3-L1 cells treated with *P. acidilactici* PGN or heat-killed *P. acidilactici* was measured using Oil Red O staining. Figure 3A shows that the treatment with *P. acidilactici* K10 PGN (K10 PGN) (10 and 20 μg/mL) reduced the lipid accumulation in 3T3-L1 cells by 23% and 18%, respectively. *P. acidilactici* HW01 PGN (HW01 PGN) also decreased the lipid accumulation in 3T3-L1 cells by 39% and 30% at concentrations of 10 and 20 μg/mL (Figure 3B). However, *P. acidilactici* PGN did not dose-dependently reduce the lipid accumulation. In a separate experiment, 10^8^ colony forming unit (CFU) per mL of heat-killed *P. acidilactici* K10 moderately inhibited the lipid accumulation, whereas 10^7^ CFU/mL of heat-killed *P. acidilactici* K10 failed to inhibit the lipid accumulation (Figure 3C). Similarly, heat-killed *P. acidilactici* HW01 inhibited the lipid accumulation at 10^8^ CFU/mL, but not at 10^7^ CFU/mL (Figure 3D). Although *P. acidilactici* PGN and heat-killed *P. acidilactici* reduced the lipid accumulation, the inhibitory effects of both postbiotic molecules, PGN and heat-killed bacteria, were not as effective compared with BLs.

### 2.3. P. acidilactici BLs Downregulate PPARγ and C/EBPα during the Adipogenesis of 3T3-L1 Cells

**Figure 4:**
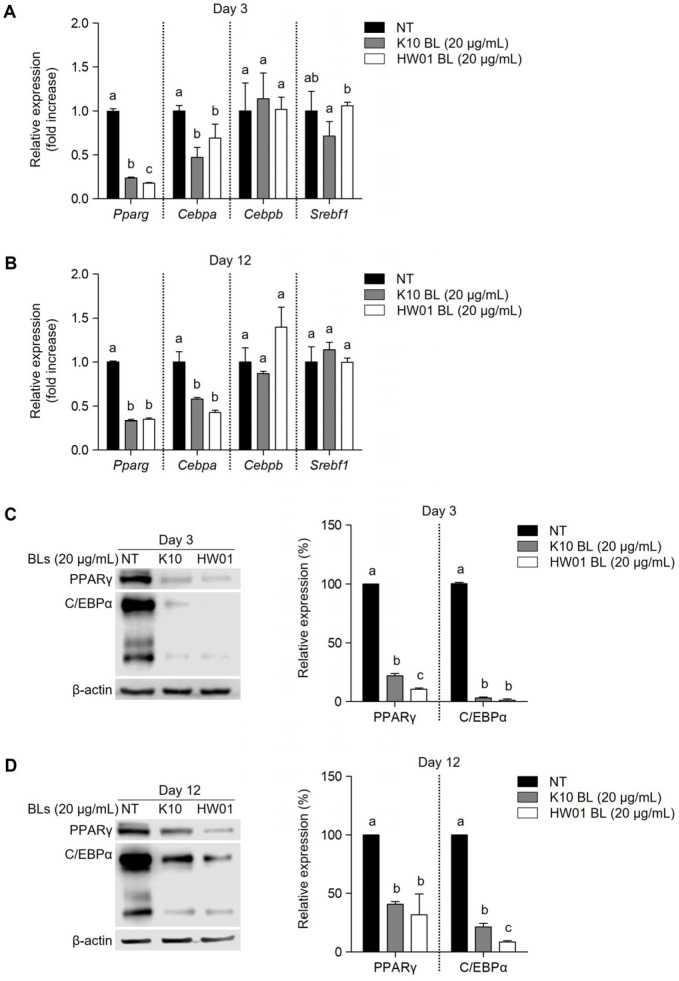
Inhibitory effect of *P. acidilactici* BLs on the expressions of adipogenic transcription factors in 3T3-L1 cells. Cells were treated with 20 μg/mL of *P. acidilactici* K10 BL (**A**) or *P. acidilactici* HW01 BL (**B**). At days 3 and 12, the mRNA expressions of *Pparg*, *Cebpa*, *Cebpb*, and *Srebf1* genes were measured by real-time quantitative reverse-transcription PCR. Cells were treated with 20 μg/mL of *P. acidilactici* K10 BL (**C**) or *P. acidilactici* HW01 BL (**D**). At days 3 and 12, cell lysates were collected and subjected to Western blot analysis to determine the protein levels of PPARγ and C/EBPα. Data are expressed as the mean ± standard deviation. Statistical significance between groups was determined by ANOVA when *p* < 0.05. Significant differences between treatment with K10 BL or HW01 BL are expressed with different letters (a, b, c). NT denotes not treated.

Because the differentiation of 3T3-L1 cells is associated with the activation of transcription factors, including PPARγ, C/EBPα, C/EBPβ, and SREBP-1c, the regulation of these transcription factors by treatment with *P. acidilactici* BLs was examined. The mRNA expressions of the *Pparg* and *Cebpa* genes were significantly inhibited in the presence of K10 or HW01 BLs (*p* < 0.05), whereas the mRNA expressions of the *Cebpb* and *Srebf1* genes were not inhibited in the presence of K10 or HW01 BLs (*p* > 0.05) at day 3 (Figure 4A). A similar phenomenon was observed on day 12. Both K10 and HW01 BLs significantly inhibited the mRNA expressions of the *Pparg* and *Cebpa* genes (*p* < 0.05) but did not inhibit the mRNA expressions of the *Cebpb* and *Srebf1* genes (*p* > 0.05) (Figure 4B). Furthermore, exposure to the K10 and HW01 BLs markedly reduced the protein levels of PPARγ and C/EBPα at day 3 and day 12 (*p* < 0.05) (Figure 4C,D, respectively). These results suggest that *P. acidilactici* BLs inhibit the differentiation of 3T3-L1 cells by downregulating two transcription factors, PPARγ and C/EBPα. During adipogenesis, C/EBPβ and C/EBPδ are expressed to induce PPARγ and C/EBPα [15,33]. Although C/EBPβ and C/EBPδ are necessary for the development of adipogenesis, the roles of these two transcription factors have not been fully elucidated [34]. The current study indicated that C/EBPβ was not regulated by *P. acidilactici* BLs. However, PPARγ and C/EBPα were considerably diminished at the mRNA and protein levels. It is generally accepted that PPARγ and C/EBPα are the master regulators of adipogenesis according to extensive studies using in vitro and in vivo models [35,36,37]. Consistent with previous studies, *P. acidilactici* BLs also downregulated PPARγ and C/EBPα, which may lead to the inhibition of adipogenesis. C/EBPβ is known to be transiently induced at the early stage of adipocyte differentiation that leads to the upregulation of late adipogenic transcription factors, such as PPARγ and C/EBPα [12]. A previous report showed that the treatment of 3T3-L1 cells with all-trans retinoic acid significantly suppresses mRNA expressions of PPARγ and C/EBPα, but not C/EBPβ. However, mRNA expression of C/EBPβ in 3T3-L1 cells after treatment with all-trans retinoic acid is transiently downregulated [38]. Moreover, the gene expression of PPARγ is inhibited, whereas that of C/EBPβ is rather enhanced in 3T3-L1 cells treated by 6-ehtoxyzolamide [39]. This can be possibly explained by C/EBPβ being regulated at the very early stage of 3T3-L1 cell differentiation. SREBP-1c is another important transcription factor that induces adipogenesis and can regulate the expressions of several genes involved in lipid metabolism [40]. Despite the importance of SREBP-1c for the development of adipogenesis, SREBP-1c did not seem to be involved in the inhibition of adipogenesis by *P. acidilactici* BLs in the current study. Overall, our results demonstrated that *P. acidilactici* BLs downregulated the expressions of PPARα and C/EBPα at the early and late stages of adipogenesis, contributing to the inhibition of 3T3-L1 cell differentiation.

### 2.4. P. acidilactici BLs Suppress Adipocyte-Specific Genes in 3T3-L1 Cells

**Figure 5:**
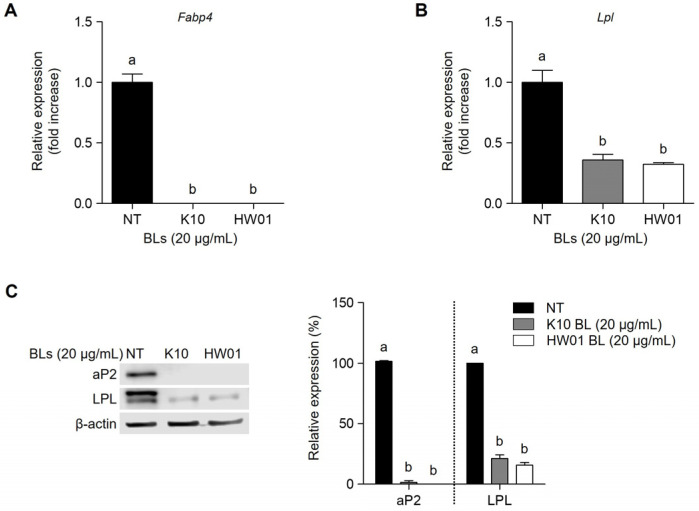
Inhibitory effect of *P. acidilactici* BLs on aP2 and LPL in 3T3-L1 cells. Cells were treated with 20 μg/mL of *P. acidilactici* K10 BL or *P. acidilactici*. At day 12, the mRNA expressions of *Fabp4* (**A**) and *Lpl* (**B**) genes were measured by real-time quantitative reverse-transcription PCR. (**C**) At day 12, cell lysates were collected and subjected to Western blot analysis to determine the protein levels of aP2 and LPL. Data are expressed as the mean ± standard deviation. Statistical significance between groups was determined by ANOVA when *p* < 0.05. Significant differences between treatment with K10 BL or HW01 BL are expressed with different letters (a, b). NT denotes not treated.

PPARγ and C/EBPα coordinately facilitate the expression of adipocyte-specific factors, such as aP2 and lipoprotein lipase (LPL), which are associated with lipid metabolism [41]. Thus, the inhibition of ap2 and LPL mRNA expressions in the presence of *P. acidilactici* BLs was examined. As shown in Figure 5A, the mRNA expression of *Fabp4* gene was significantly decreased (*p* < 0.05) when 3T3-L1 cells were treated with 20 μg/mL of K10 or HW01 BL. Moreover, both K10 and HW01 BL (20 μg/mL) significantly downregulated *Lpl* gene expression in 3T3-L1 cells (*p* < 0.05) (Figure 5B). We further examined the protein levels of ap2 and LPL in 3T3-L1 cells after treatment with *P. acidilactici* BLs. As expected, the protein levels of aP2 and LPL were markedly reduced by treatment with K10 or HW BL (20 μg/mL) (Figure 5C). aP2, also known as fatty acid-binding protein 4, is involved in the intracellular transport and metabolism of fatty acids during adipogenesis [42]. Triglycerides are hydrolyzed by LPL, promoting the uptake of fatty acids by the surrounding tissues [43]. Previous studies showed that the reduced expression of PPARγ resulted in the decreased mRNA and protein levels of aP2 and LPL [14,44]. Consistently, our findings also demonstrated that *P. acidilactici* BLs suppressed the expressions of aP2 and LPL by regulating PPARγ and C/EBPα. These results suggest that *P. acidilactici* BLs reduced aP2 and LPL expressions, which may impair lipid accumulation during the differentiation of 3T3-L1 cells.

### 2.5. P. acidilactici BLs Reduces Adipokines in 3T3-L1 Cells

**Figure 6:**
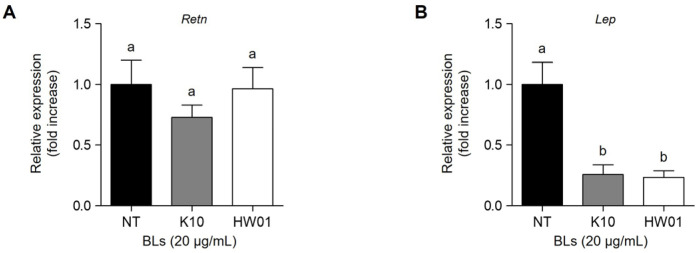
Inhibitory effect of P. acidilactici BLs on the mRNA expressions of adipokines in 3T3-L1 cells. Cells were treated with 20 μg/mL of *P. acidilactici* K10 BL or *P. acidilactici* HW01 BL. At day 12, the mRNA expressions of *Retn* (**A**) and *Lep* (**B**) genes were measured by real-time quantitative reverse-transcription PCR. Data are expressed as the mean ± standard deviation. Statistical significance between groups was determined by ANOVA when *p* < 0.05. Significant differences between treatment with K10 BL or HW01 BL are expressed with different letters (a, b). NT denotes not treated.

Adipokines, such as resistin and leptin, are cytokines secreted from adipocytes [45]. Resistin is preferentially expressed in adipose tissue and is associated with type 2 diabetes and metabolic syndrome [46,47]. Thus, we also examined whether *P. acidilactici* BLs inhibited mRNA expression in 3T3-L1 cells. Although K10 BL slightly inhibited the mRNA expression of the resistin gene (*Retn*), the inhibition by K10 BL was not statistically significant (*p* > 0.05). Similarly, HW01 BL did not inhibit the mRNA expression of *Retn* gene (Figure 6A). However, when 3T3-L1 cells were treated with K10 or HW01 BLs, the mRNA expression of *Lep* gene was significantly inhibited (*p* < 0.05) (Figure 6B). Leptin can induce the formation of lipid droplets, accelerating the differentiation of 3T3-L1 cells [48]. Thus, leptin is highly associated with obesity [49]. Accordingly, it can be assumed that the decreased leptin expression through the inhibition of PPARγ by *P. acidilactici* BLs could inhibit the differentiation of 3T3-L1 cells.

## 3. Materials and Methods

### 3.1. Microorganisms and Sample Preparation

*P. acidilactici* K10 and *P. acidilactici* HW01 were isolated from kimchi and malt, respectively [50,51]. The bacteria were cultured in Man-Rogosa-Sharpe (MRS) (Neogen, Lansing, MI, USA) at 37 °C for 24 h. In order to extract BLs, bacterial pellets were collected after centrifuging at 6000× *g* for 10 min and washed twice with phosphate-buffered saline (PBS). Then, the bacterial pellets were suspended in extraction buffer (50 mM Trizma base, 0.1 mM EDTA, and 1 mM 2-mercaptoethanol, pH 7.5), transferred to a tube containing zirconium beads (Benchmark Scientific, Sayreville, NJ, USA) and homogenized at 4 °C for 100 s using a benchtop homogenizer (BeadBug^TM^ 3, Benchmark Scientific). The supernatants were collected by centrifuging at 20,000× *g* for 30 min and filtered through a syringed filter (0.2 μm). The concentration of BLs was determined by bicinchoninic acid (BCA) protein assay (Thermo Fisher Scientific, Rockford, IL, USA). Crude PGN of *P. acidilactici* was prepared as described previously with minor modifications [52]. After culturing and harvesting *P. acidilactici*, bacterial pellets were washed with 1 M NaCl and homogenized in a tube containing zirconium beads as described above. The supernatants were discarded, and the remaining pellets were incubated with 0.5% sodium dodecyl sulfate in PBS at 60 °C for 30 min. Then, the resulting cell walls were extensively washed with distilled water five times and incubated in 50 mM Tris-HCl (pH 7.0) containing 50 μg DNase and 250 μg RNase at 37 °C for 2 h to remove residual nucleic acids followed by the addition of 50 mg MgCl_2_ and 1 mg trypsin and further incubation at 37 °C for 24 h to remove residual nucleic acids and cell wall bound proteins. After centrifugation at 19,000× *g* for 10 min, the cell walls were incubated in 48% hydrofluoric acid at 4 °C overnight to remove wall teichoic acids. After extensively washing with distilled water, insoluble crude PGN was collected and lyophilized. To prepare soluble crude PGN, 100 μg insoluble crude PGN was incubated with 50 U mutanolysin in endotoxin-free water at 37 °C for 24 h. The enzyme was inactivated by boiling for 10 min. To prepare heat-killed bacteria, *P. acidilactici* was grown in MRS broth at 37 °C for 24 h and harvested by centrifuging. The bacterial pellets were washed three times with PBS and killed by heating at 80 °C for 2 h. To ensure that the bacteria were completely killed, the heat-treated bacteria were plated on MRS agar and cultured at 37 °C for 24 h.

### 3.2. Cell Culture and Differentiation of 3T3-L1 Cells

Murine 3T3-L1 pre-adipocytes were purchased from the Korean Cell Line Bank (Seoul, Korea) and grown in Dulbecco’s modified Eagle’s medium (DMEM; Welgene, Gyeongsan, Korea) supplemented with 10% bovine calf serum, 100 U/mL penicillin, and 100 μg/mL streptomycin (HyClone, Logan, UT, USA) at 37 °C in a 5% CO_2_-humidified incubator. To differentiate 3T3-L1 pre-adipocytes, the cells were seeded in a 6-well culture plate and incubated until the cells were fully confluent. At 2 days after confluence (day 0), the culture medium was changed to differentiation medium (DMEM containing 10% fetal bovine serum, 5 μg/mL insulin, 0.5 mM 3-isobutyl-1-methylxanthine, and 1 μM dexamethasone) and incubated with *P. acidilactici* BLs (0, 5, 10, or 20 μg/mL), *P. acidilactici* PGN (0, 10, or 20 μg/mL), or heat-killed *P. acidilactici* (0, 10^7^, or 10^8^ CFU/mL) for 3 days (day 3). The spent culture medium was discarded, and the 3T3-L1 cells were further incubated with *P. acidilactici* BLs (0, 5, 10, or 20 μg/mL), *P. acidilactici* PGN (0, 10, or 20 μg/mL), or heat-killed *P. acidilactici* (0, 10^7^, or 10^8^ CFU/mL) in DMEM containing 10% fetal bovine serum and 10 μg/mL insulin for 3 days (day 6). The spent culture medium was replaced with fresh DMEM containing 10% fetal bovine serum and 10 μg/mL insulin every 3 days and incubated up to day 12.

### 3.3. Quantification of Lipid Droplets and Triglycerides in 3T3-L1 Cells

After treating 3T3-L1 cells for 12 days, the cells were carefully rinsed with PBS and fixed with 4% formaldehyde at room temperature for 1 h, followed by washing with 60% isopropanol. The 3T3-L1 cells were then stained with Oil Red O solution (Sigma-Aldrich, St. Louis, MO, USA) at room temperature for 30 min and washed with 60% isopropanol and distilled water. Stained lipid droplets were quantified at an optical density of 492 nm using a microtiter plate reader (Allsheng, Hangzhou, China). For the quantification of cellular triglycerides, a triglyceride assay kit (Abcam, Cambridge, UK) was used. Briefly, after treating 3T3-L1 cells with BLs (0, 5, 10, or 20 μg/mL), the cells were washed with PBS and resuspended in 5% NP-40, followed by boiling for 5 min. The cell suspension was centrifuged at 13,000× *g* for 2 min and the supernatants were mixed with lipases to convert triglycerides into glycerol. After adding a triglyceride probe and a triglyceride enzyme mix, the mixture was incubated at room temperature for 1 h and the optical density was measured at 570 nm using a microtiter plate reader (Allsheng). The concentration of triglycerides was determined based on the standard curve of glycerol.

### 3.4. Quantitative Reverse Transcription Polymerase Chain Reaction

Total RNA was extracted from 3T3-L1 cells treated with or without BLs at day 3 or 12 using TRIzol reagent (Invitrogen, Carlsbad, CA, USA) according to the manufacturer’s instructions and transcribed to complementary DNA (cDNA) using random hexamers and reverse transcriptase (Promega, Madison, WI, USA) in a total volume of 30 μL. In order to amplify cDNA, real-time quantitative reverse transcription polymerase chain reaction (qRT-PCR) was performed with SYBR Green Real-Time PCR master mix (Toyobo, Osaka, Japan) using the StepOnePlus^TM^ real-time PCR system (Applied Biosystems, Foster City, CA, USA). The amplification conditions were as follows: an initial denaturation at 95 °C for 10 s, and amplification for 40 cycles at 95 °C for 5 s, and at 60 °C for 31 s. The sequences of the PCR primers used in this study were as follows: *Pparg* (PPARγ), forward 5′-AAGGGTGCCAGTTTCGATCC-3′ and reverse 5′-TCCTTGGCCCTCTGAGATGA-3′; *Cebpa* (CEBP/α), forward 5′-GAGACCGAGAGACTTTCCGC-3′ and reverse 5′-TCATTTTTCTCACGGGGCCA-3′; *Cebpb* (CEBP/β), forward 5′-GCTGAGCGACGAGTACAAGAT-3′ and reverse 5′-CAGCTGCTTGAACAAGTTCCG-3′; *Srebf1* (SREBP-1c), forward 5′-CTCAGCAGCCCCTAGAACAAA-3′ and reverse 5′-ATGGTCCCTCCACTCACCA-3′; *Fabp4* (adipocyte fatty acid-binding protein, aP2), forward 5′-GCCCAACATGATCATCAGCG-3′ and reverse 5′-TGGTCGACTTTCCATCCCAC-3′; *Lpl* (lipoprotein lipase), forward 5′-AACATTCCCTTCACCCTGCC-3′ and reverse 5′-GTCTCTCCGGCTTTCACTCG-3′; *Retn* (resistin), forward 5′-AATCCTCCCTTCTGCAGTTCC-3′ and reverse 5′-AGTCTGGGAGGGAGTCCTAAG-3′; *Lep* (leptin), forward 5′-CTATGCCACCTTGGTCACCT-3′ and reverse 5′-ACCAAACCAAGCATTTTTGC-3′; and *ACTB* (β-actin), forward 5′-TACAGCTTCACCACCACAGC-3′ and reverse 5′-GGAAAAGAGCCTCAGGGCAT-3′. The relative mRNA expressions of specific genes were normalized to β-actin by the 2^−∆∆Ct^ method.

### 3.5. Western Blot Analysis

Lysates of 3T3-L1 cells treated with or without BLs at day 3 or 12 were obtained using lysis buffer (1 M HEPES, pH 7.5, 1 M NaCl, 1% IGEPAL^®^-CA630, 0.75% sodium deoxycholate, 10% glycerol) supplemented with proteinase inhibitors. The protein concentration of lysates was measured by BCA assay (Thermo Fisher Scientific). Equal amounts of proteins (20 μg) were separated by sodium dodecyl sulfate-polyacrylamide gel electrophoresis and transferred to polyvinylidene difluoride (PVDF) membranes (Millipore, Bedford, MA, USA). After blocking with 5% skimmed milk, the PVDF membranes were probed with primary antibodies against PPARγ, C/EBPα (Cell Signaling Technology, Danvers, MA, USA), aP2, LPL, and β-actin (Santa Cruz Biotechnology, CA, USA) at 4 °C overnight. The specific proteins were detected using horseradish peroxidase-conjugated anti-rabbit IgG antibody (Cell Signaling Technology) or anti-mouse IgG antibody (Santa Cruz Biotechnology) by incubation with the PVDF membranes at room temperature for 1 h. The immunoreactive proteins were visualized with an enhanced chemiluminescence reagent (Dyne Bio, Seongnam, Korea) and quantified using a C-DiGit Blot scanner (Li-Cor Bioscience, Lincoln, NE, USA).

### 3.6. Statistical Analysis

All data presented in this study were obtained from three independent experiments. Each experiment was carried out at least three times. The results are expressed as the mean ± standard deviation. Statistical significance between treatment and control groups was determined one-way analysis of variance (ANOVA) when *p* < 0.05 using IBM SPSS Statistics 23 software (IBM, Armonk, NY, USA).

## 4. Conclusions

In summary, the current study showed that *P. acidilactici* BLs reduced the differentiation of 3T3-L1 cells by reducing the formation of lipid droplets and cellular triglycerides. *P. acidilactici* BLs also inhibited the master transcription factors responsible for adipogenesis, PPARγ and C/EBPα, leading to the reduction of 3T3-L1 cell differentiation. In addition, the expressions of lipogenic genes, such as aP2 and LPL, and adipokines, such as leptin, were inhibited by *P. acidilactici* BLs. Although further studies are needed to elucidate whether *P. acidilactici* BLs regulate the body weight and body fat mass in vivo models, the current study provides evidence that *P. acidilactici* BLs might be potential anti-obesity agents.

## Data Availability

The data in this study can be requested from the corresponding author.
